# Draft genome sequence data of plant growth promoting and calcium carbonate precipitating *Bacillus velezensis* CMU008

**DOI:** 10.1016/j.dib.2023.108965

**Published:** 2023-02-09

**Authors:** Pharada Rangseekaew, Natnaree Ua-arak, Wasu Pathom-aree

**Affiliations:** a Research Center of Microbial Diversity and Sustainable Utilization, Department of Biology, Faculty of Science, Chiang Mai University, Chiang Mai 50200, Thailand; b International Community School, Bangkok 10260, Thailand

**Keywords:** Bacillus, Draft genome sequence, Illumina, Calcium carbonate precipitation, Plant growth promoting bacteria

## Abstract

A Gram-positive, spore forming bacterium designated as strain CMU008 was isolated from a soil sample in Chiang Mai University campus, Chiang Mai, Thailand. This strain is able to precipitate calcium carbonate and promote growth of sunflower sprouts. The whole genome sequencing was done using Illumina MiSeq platform. The draft genome of strain CMU008 was 4,016,758 bp in length with 4,220 protein coding sequences and an average G + C content of 46.01 mol%. The ANIb values of strain CMU008 and the type strains of its closely related neighbors, *Bacillus velezensis* NRRL B-41580^T^ and *B. velezensis* KCTC13012^T^ were 98.52%. Phylogenomic tree also supports the assignment of strain CMU008 as *B. velezensis*. The genome sequence data of *B. velezensis* strain CMU008 provide insightful information for the taxonomic characterization and further biotechnological exploitation of this strain. The draft genome sequence data of *B. velezensis* strain CMU008 has been deposited in the DDBJ/EMBL/GenBank databases under the accession number JAOSYX000000000.

**Specifications Table**

**Table:** 

Subject	*Biology*
Specific subject area	*Microbiology, genomics*
Type of data	Table Figure Draft genome sequence
How the data were acquired	Genome sequencing was performed on Illumina MiSeq Sequencer at Omics Science and Bioinformatics Center, Faculty of Science, Chulalongkorn University, Bangkok, Thailand
Data format	Raw, analyzed and assembled genome sequence
Description of data collection	A pure culture of *Bacillus velezensis* CMU008 was routinely cultured on tryptic soy agar (TSA) at 37 °C. Genomic DNA was extracted from a 24 h culture on TSA and used as template for sequencing reaction
Data source location	*• Institution: Chiang Mai University* *• City/Town/Region: Chiang Mai* *• Country: Thailand* *• Latitude and longitude:* 18.48 N 98.57 E
Data accessibility	The complete genome sequence of *Bacillus velezensis* CMU008 was deposited in NCBI GenBank under accession number JAOSYX000000000 Direct URL to data: https://www.ncbi.nlm.nih.gov/bioproject/886473 Database link: BioProject: PRJNA886473 BioSample: SAMN31133207

## Value of the Data

• The draft genome of *Bacillus velezensis* CMU008 can provide insights for the understanding of calcium carbonate precipitation and plant growth promotion potential.
• These data are valuable resources for researchers working in the field of Microbiology, Genomics, and Molecular Biology.
• This genome data can be used for comparative genomics of members of the genus *Bacillus* for biotechnological and taxonomic purposes and allow in depth analysis of *Bacillus velezensis* CMU008 via genome mining.

## 1 Objectives

The generation of draft genome sequence of *Bacillus velezensis* CMU008 aims to identify protein encoding genes that are responsible for calcium carbonate precipitation and plant growth promotion. The identification of such genes in *B. velezensis* CMU008 genome is important for the understanding of molecular mechanisms that can support calcium carbonate precipitating and plant growth promoting abilities of this bacteria for further applications.

## 2 Data Description

*Bacillus* sp. strain CMU008 was isolated from garden soil collected from Chiang Mai University, Chiang Mai, Thailand (18.48 N 98.57 E). Top soil (5 cm depth) was collected using sterile spoon. The strain was isolated by dilution spread plate on nutrient agar. Strain CMU008 was able to precipitate calcium carbonate (CaCO_3_) via the production of urease enzyme. *Bacillus* sp. strain CMU008 exhibited ability to promote the growth of sunflower sprout and increase CaCO_3_ precipitation in soil. Here we report the genome sequence of *Bacillus* sp. strain CMU008 to facilitate further study of gene related to CaCO_3_ precipitation and plant growth promotion.

Table 1 summarized the genome characteristics of *Bacillus* sp. strain CMU008. The draft genome contains 52 contigs with genome length of 4016,758 bp, N50 and L50 value of 174, 703 and 7, respectively. The genome contains 4220 protein coding sequence (CDS), 75 tRNAs genes, 3 rRNA genes with 46.01 G + C content (%). The data have been deposited in GenBank and can be viewed at https://www.ncbi.nlm.nih.gov/bioproject/886473

**Table 1:** Genome characteristics of *Bacillus* sp. strain CMU008.

Features	Value
Number of contigs	52
Genome length	4016,758 bp
Genome coverage	115X
Largest contig	534,212
GC (%)	46.01
Plasmid	0
N50	174, 703
L50	7
Protein coding sequence (CDS)	4220
tRNA	74
rRNA	3

**Fig. 1:**
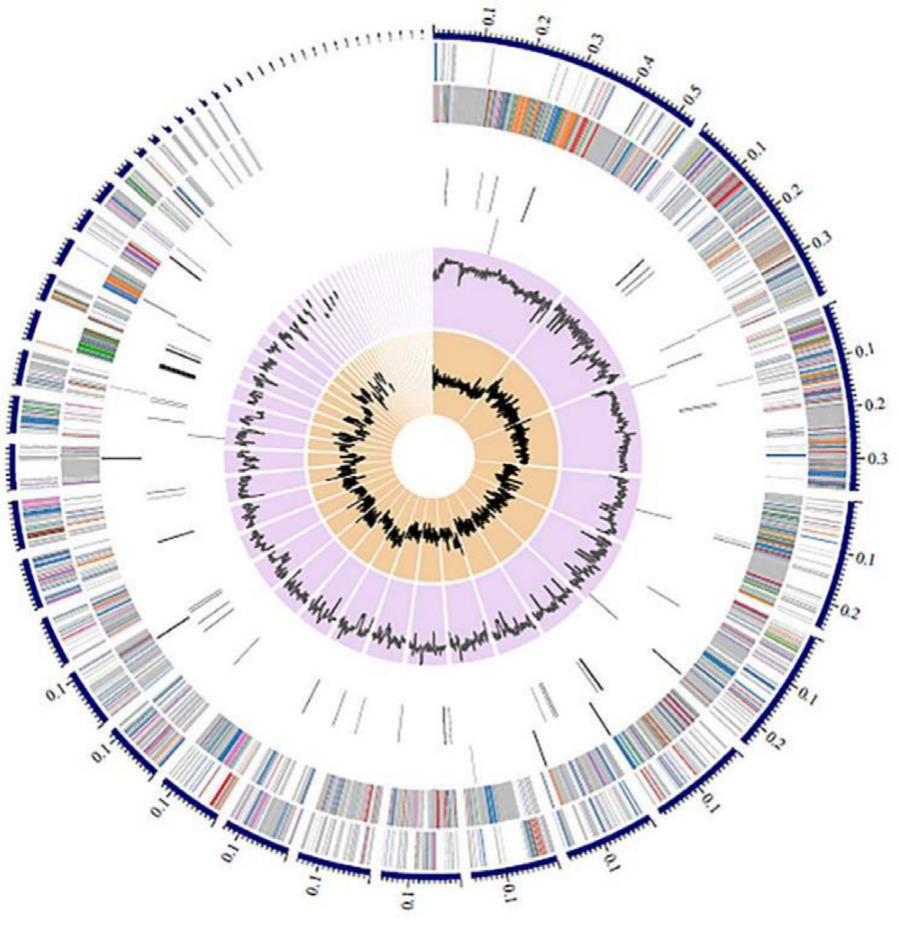
The distribution of annotated genomic features. This includes, from outer to inner rings, the contigs, CDS on the forward strand, CDS on the reverse strand, RNA genes, CDS with homology to know antimicrobial resistance genes, CDS with homology to known virulence factors, GC content and GC skew. The color of the CDS on the forward and reverse strand indicates the subsystem that these genes belong to (Fig. 2).

**Fig. 2:**
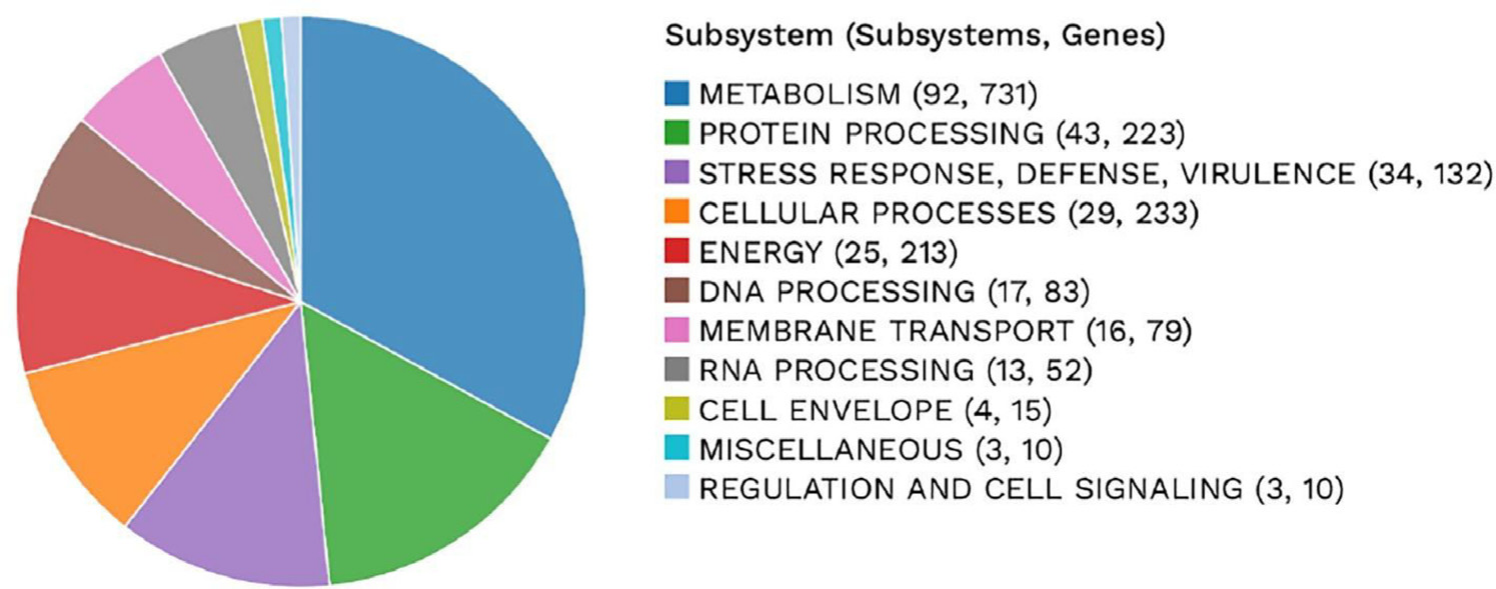
PATRIC annotation using RAST tool kit (RASTtk) of *Bacillus* sp. strain CMU008’s genome.

The annotated genome of *Bacillus* sp. strain CMU008 was analyzed using the PATRIC genome analysis server (https://www.patricbrc.org/) [1] to identify the genome and protein features which revealed two types of protein families [2]. The 3899 proteins belong to genus-specific protein families (PLFams) and 4024 proteins belong to the cross-genus protein families (PGFams)*.* A circular map of *Bacillus* sp. strain CMU008 genome presents the distribution of genome annotation (Fig. 1). Fig. 2 displays the subsystem of proteins which were categorized into 239 subsystems with 11 biological processes. The number of genes assigned to each biological processes is as followed: metabolism (731), cellular processes (233), protein processing (223), energy (213), stress response, defense and virulence (132), DNA processing (83), membrane transport (79), RNA processing (52), cell envelop (15), miscellaneous (10) and regulation and cell signaling (10).

The genome annotation of *Bacillus* sp. strain CMU008 sequence in PATRIC uses k-mer-based antimicrobial resistance (AMR) genes detection method which utilizes PATRIC's curated collection of representative AMR gene sequence variants [1]. Each AMR gene functional annotation, broad mechanism of antibiotic resistance, drug class and, in some cases, specific antibiotic it confers resistance to, are shown in Table 2.

**Table 2:** Antimicrobial resistance (AMR) genes.

AMR mechanism	Genes
Antibiotic inactivation enzyme	ANT(6)-I, BcII family, FosB
Antibiotic target in susceptible species	Alr, Ddl, dxr, EF-G, EF-Tu, folA, Dfr, folP, gyrA, gyrB, inhA, fabI, Iso-tRNA, kasA, MurA, rho, rpoB, rpoC, S10p, S12p
Antibiotic target modifying enzyme	Cfr, RlmA(II)
Antibiotic target protection protein	BcrC
Antibiotic target replacement protein	FabL
Efflux pump conferring antibiotic resistance	BceA, BceB, EbrA, EbrB, Lmr(B), Tet(L), YkkCD
Gene conferring resistance via absence	gidB
Protein altering cell wall charge conferring antibiotic resistance	GdpD, MprF, PgsA
Regulator modulating expression of antibiotic resistance gene	BceR, BceS, LiaF, LiaR, LiaS

**Table 3:** ANIb values of *Bacillus* sp. strain CMU008 with its closely related *Bacillus* spp.

Genome	ANIb (%)
*Bacillus velezensis* KTCT 13012^T^	98.52
*Bacillus velezensis* NRRL B-41580^T^	98.52
*Bacillus velezensis* NRRL B-4257	98.47
*Bacillus amyloliquefaciens* Bs006	97.64
*Bacillus subtilis* B-1	97.42
*Bacillus siamensis* KTCT 13613^T^	93.71

**Fig. 3:**
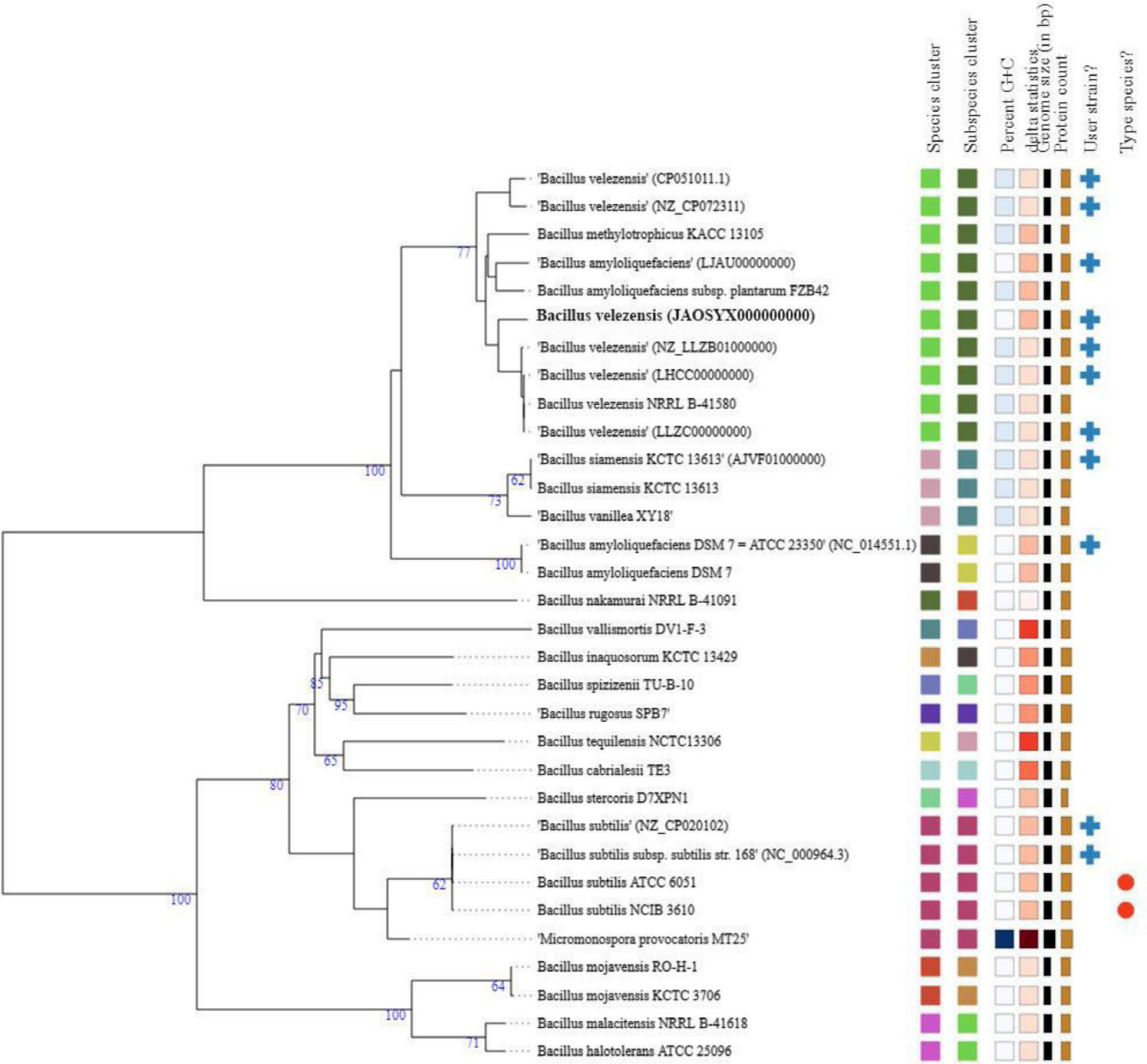
Phylogenomic tree of *Bacillus* sp. strain CMU008 and its closely related *Bacillus* spp. genome generated using the Type (Strain) Genome Server (TYGS).

The phylogenomic tree showed that *Bacillus* sp. strain CMU008 formed clade with several *B. velezensis* including type strain of *B. velezensis* NRRL B-41580^T^ (LLZC00000000) and *B. velezensis* KCTC13012^T^ (LHCC00000000) (Fig. 3). Additionally, whole-genome comparisons using JSpeciesWS web server tool, revealed the average nucleotide identity (ANIb) values between *Bacillus* sp. strain CMU008 and type strains of *B. velezensis* NRRL B-41580^T^ and *B. velezensis* KCTC13012^T^ were 98.52%. These data confirmed the assignment of strain CMU008 as *B. velezensis* strain CMU008.

## 3 Experimental Design, Materials and Methods

### 3.1 Bacterial cultivation and genomic DNA extraction

*Bacillus velezensis* CMU008 was cultured on tryptic soy agar (TAS) at 37 °C for 24 h. Genomic DNA extraction was performed by the following procedures. Bacterial cells were lysed by extraction buffer. Phenol was added to remove protein and centrifuged at 15,000 rpm for 5 min at 4 °C. The supernatant was repeated for phenol extraction step. To precipitate DNA, sodium acetate, isopropanol and absolute ethanol were added and incubated at −20 °C for 15 min. After incubation, precipitated DNA was harvested by centrifugation at 15,000 rpm for 5 min at 4 °C. Then, DNA was washed by 70% (v/v) ethanol and centrifuged at 15,000 rpm for 5 min at 4 °C. DNA was dried for 30 min and dissolved with sterile ultrapure water.

### 3.2 Whole genome sequencing, assembly annotation and analysis

The genomic DNA of *Bacillus velezensis* CMU008 was sequenced using service of Omics Science and Bioinformatics Center, Faculty of Science, Chulalongkorn University, Bangkok, Thailand. The genomic DNA library was prepared using QIASEQ FX DNA library preparation kit (Qiagen, USA). The libraries were sequenced on Illumina MiSeq sequencer in 2 × 250 bp paired end. Raw reads quality was checked using FASTQC software version 0.11.9 [3]. Adaptors and poor-quality reads were removed using Fastp version 0.23.2 [4], and the filtered reads were used as an input for Unicycler, genome assembly program [5]. Annotation of assembled genome was done using the PATRIC RASTtk-enabled Genome Annotation Service [6]. In addition, ANIb was calculated and compared using JSpeciesWS version: 3.9.7, web server tool [7]. Phylogenomic tree was constructed using the Type (Strain) Genome Server (TYGS) [8,9] (https://tygs.dsmz.de/). All software was run with default parameters.

## Ethics Statements

This study did not involve any human subjects and animal experiments. No ethical approval was required.

## CRediT authorship contribution statement

**Pharada Rangseekaew:** Conceptualization, Methodology, Investigation, Formal analysis, Writing – review & editing. **Natnaree Ua-arak:** Investigation, Formal analysis. **Wasu Pathom-aree:** Conceptualization, Formal analysis, Supervision, Writing – review & editing.

## Declaration of Competing Interest

The authors declare that they have no known competing financial interests or personal relationships that could have appeared to influence the work reported in this paper.

## Data Availability

Bacillus velezensis strain CMU008 (Original data) (GenBank).
